# Successful limb salvage through staged bypass combined with free gracilis muscle transfer for critical limb ischemia with osteomyelitis after failed endovascular therapy

**DOI:** 10.1186/s40792-018-0449-9

**Published:** 2018-05-02

**Authors:** Keisuke Miyake, Shinsuke Kikuchi, Hiroko Okuda, Atsuhiro Koya, Satomi Abe, Yoshiki Sawa, Tetsuo Ota, Nobuyoshi Azuma

**Affiliations:** 1 Department of Vascular Surgery, Asahikawa Medical University, 1-1-1, Midorigaoka-higashi-2jyo, Asahikawa, 078-8510 Japan; 2 Department of Cardiovascular Surgery, Graduate School of Medicine, Osaka University, Osaka, Japan; 3 Department of Orthopedic Surgery, Asahikawa Medical University, Asahikawa, Japan; 4 Department of Physical Medicine and Rehabilitation, Asahikawa Medical University Hospital, Asahikawa, Japan

**Keywords:** Critical limb ischemia, Osteomyelitis, Gracilis muscle flap, Surgical bypass, Failed endovascular therapy

## Abstract

**Background:**

Critical limb ischemia with osteomyelitis is so difficult to treat that even appropriate revascularization and wound therapy cannot achieve limb salvage because of uncontrollable infection. It is still difficult to judge the possibility of limb salvage before revascularization.

**Case presentation:**

A 73-year-old male complained of a small ulcer on his left toe, which was treated with multiple endovascular therapy. After failed endovascular therapy, he suffered extensive tissue loss with tibial osteomyelitis. We carried out staged surgery that was composed of dual bypass to the sural artery and posterior tibial artery. After intensive debridement and wound care, insertion of a subsequent free gracilis muscle flap to cover the exposed tibial bone was performed, achieving functional limb salvage.

**Conclusion:**

Even in the threatened limb with extensive tissue loss and osteomyelitis, intensive and multidisciplinary treatment with staged revascularization, muscle transfer, and appropriate wound care achieved functional limb salvage.

## Background

Critical limb ischemia (CLI) complicated with osteomyelitis is difficult to treat even after appropriate revascularization and antibiotic therapies. Osteomyelitis is diagnosed by physical examination, laboratory values, plain X-ray film, and magnetic resonance imaging (MRI) findings. Of these, MRI is the most accurate imaging modality for detecting osteomyelitis [1]. However, even such an accurate imaging modality cannot predict the curability of osteomyelitis; it cannot be determined whether conservative therapy is enough, some intensive treatment is required, or infected bone removal is mandatory.

In the setting of CLI with major tissue loss and infection, restoring sufficient blood flow to the wound is particularly important. In this regard, bypass surgery is more suitable to treat CLI with major tissue loss or infection than endovascular therapy (EVT) [2].

Here, we report successful limb salvage in a case of major tissue loss with severe infection, in which we had difficulty in judging whether limb salvage was possible. Limb salvage was achieved by staged surgical revascularization combined with free gracilis muscle transfer to cover an exposed tibial bone that was affected by osteomyelitis.

## Case presentation

**Fig. 1:**
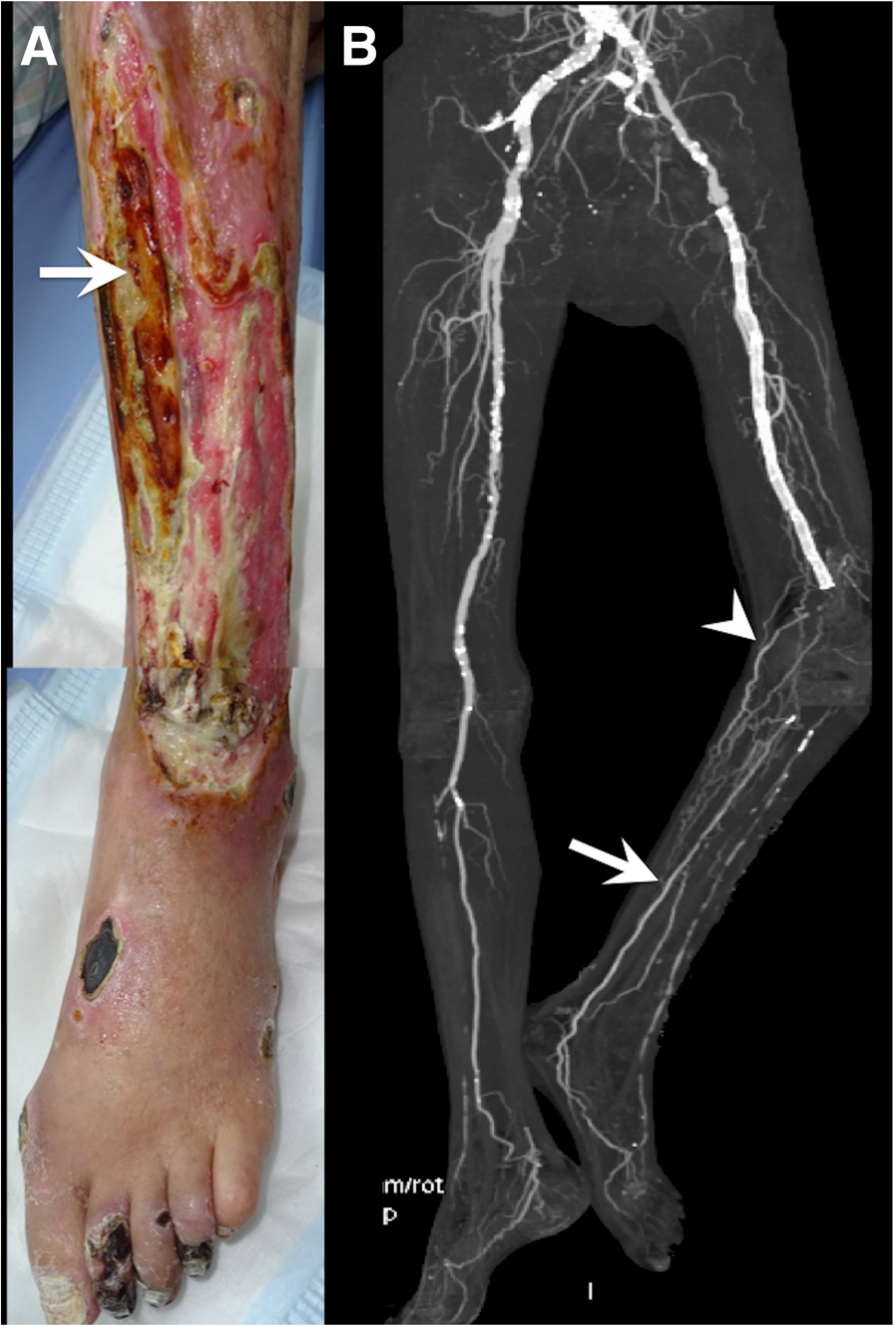
**a** Preoperative photograph showing the severe tissue defect at the lower leg in the form of an exposed tibia (arrow), multiple ulcers, and necrosis of the foot, including the toe, dorsum, lateral malleolus, and heel. **b** Preoperative computed tomography showed multiple arterial lesions, a patent posterior tibial artery (arrow), and well-developed sural artery (arrowhead)

A 73-year-old male with a past medical history of hypertension complained of a small ulcer on his left toe; examination revealed CLI. Cardiologists performed multiple EVTs, including stenting the superficial femoral artery (SFA) as well as balloon angioplasty in the common femoral artery (CFA), deep femoral artery (DFA), and tibial arteries. After EVT of the anterior tibial artery (ATA), the ATA ruptured and compartment syndrome of the calf subsequently occurred. Fasciotomy and inappropriate debridement were performed and led to major tissue loss with consequent exposure of the anterior tibial surface (Fig. 1a) because of persistent limb ischemia, even after multiple EVTs. The patient was advised to have a major amputation, but he did not want this, and he came to our hospital seeking a second opinion. Bacterial culture of the wound showed co-infection of *Staphylococcus aureus* and Enterobacter. Initially, the likelihood of limb salvage seemed to be low because of the extensive tissue loss with osteomyelitis of the exposed tibia. However, we decided to seek the possibility of limb salvage considering the following conditions: (1) the patient was able to walk under cane support and eagerly wanted to maintain ambulatory status and (2) the patient had no atherosclerotic risk factors other than hypertension without significant comorbidities. The prognosis seemed to be good once limb salvage was achieved.

Computed tomography angiography (CTA) showed stenosis in the external iliac artery (EIA), total occlusion of the SFA and peroneal artery, severely diseased ATA, patent posterior tibial artery (PTA), and well-developed sural artery (SA) (Fig. 1b), revealing the PTA as an ideal target for bypass surgery. However, there was a considerable risk of major amputation even after bypass surgery in this patient because of osteomyelitis in his tibia and tarsal bones. If major amputation was inevitable even after bypass surgery to the disease-free distal PTA, the bypass graft would be sacrificed and the amputation level would be high, that is, above the knee level because of the following persistent ischemia around the knee area. Therefore, we decided to perform dual bypass surgery to supply blood flow to both around the knee and the tissue-defect area; this surgery involved a bypass to the SA and to the proximal portion of the PTA. The possibility of the limb salvage was unclear in this point, and only when the granulation process was satisfactory and infection around the wound and osteomyelitis were well controlled, additional procedures including more distal bypass and free tissue transfer for limb salvage would be planned.

In the first operation, after dissecting the femoral arteries from the longitudinal inguinal incision, EIA stenting was performed via the CFA, followed by patch angioplasty from the CFA to the DFA with a saphenous vein as the inflow reconstruction. The SA, which was mapped preoperatively by duplex imaging, was dissected through a direct approach based on its anatomic location. A saphenous vein branch with a relatively large caliber was anastomosed to the dissected SA with 8/0 polypropylene sutures. Then, the main trunk of the saphenous vein was anastomosed to the proximal PTA with 8/0 polypropylene sutures in an in situ fashion. Completion angiography detected no abnormality. We chose the proximal PTA as the distal anastomosis site, because of the extensive infection involving the middle part of the tibia. If we selected the more distal disease-free PTA as the anastomotic site, in case the infection around the wound is uncontrolled and extending, there was a considerable risk of graft infection and subsequent graft rupture.

**Fig. 2:**
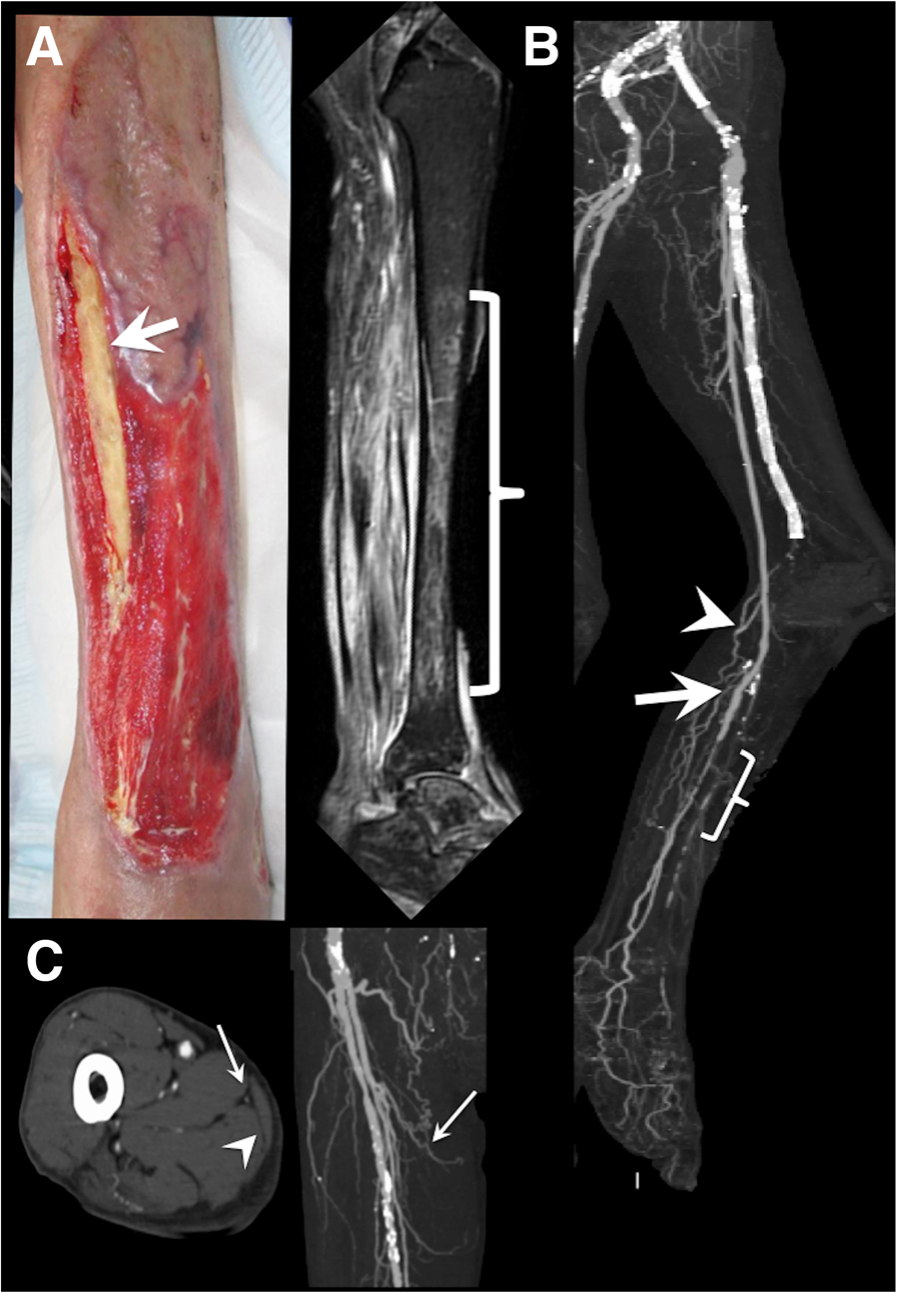
**a** Two months after initial surgery. A photograph showing the exposed surface of the tibia even with good granulation around the area. Magnetic resonance imaging showed osteomyelitis of the tibia (bracket). **b** Postoperative computed tomography showed patent bypasses to the sural artery (arrowhead) and posterior tibial artery (arrow). Native artery stenosis was detected distal to the anastomosis (bracket). **c** The gracilis muscle (arrowhead) and the pedicle (arrow) of the contralateral leg that originated from the medial femoral circumflex artery

Following the operation, the granulation process became marked, but the wound in front of the tibia was refractory; MRI detected chronic osteomyelitis of the exposed tibia. Two months after the initial surgery, the granulation covered most of the crural and foot ulcers but was not sufficient to fully cover the exposed tibia (Fig. 2a). Postoperative CTA, 2 months after surgery, revealed native PTA stenosis distal to the distal anastomosis site, the restenotic lesion after the previous EVT (Fig. 2b), though the stenosis was not so severe to threaten the graft patency in duplex ultrasound. Systemic inflammation and infection were well-controlled by antibiotic infusion which was initially vancomycin hydrochloride and changed to daptomycin; a blood test showed the white blood cell count to be 8240/μl and the C-reactive protein concentration to be 1.33 mg/dl. From these findings, which showed a low-grade inflammation level, we judged that the tibial osteomyelitis could be cured if we could successfully cover the exposed bone surface with a muscle flap. Then, we decided to perform a second operation to achieve limb salvage with a tissue transfer, combined with an additional bypass to supply the inflow site for tissue transfer and more blood flow to the foot.

**Fig. 3:**
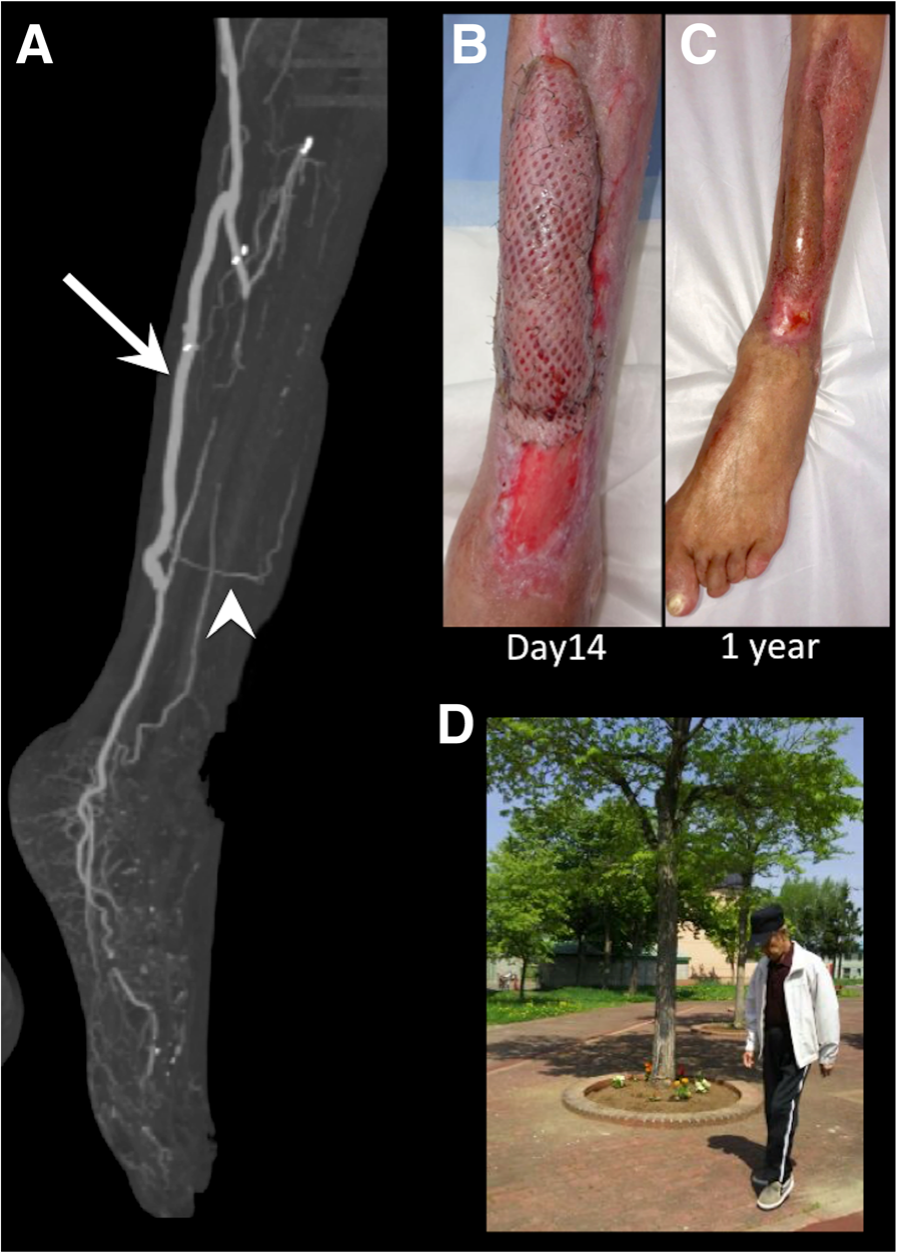
**a** Computed tomography after the second surgery showed the patent jump bypass to the distal posterior tibial artery (arrow) and the pedicle of the free gracilis muscle flap (arrowhead). **b** Postoperative photographs showing the gracilis muscle flap covered with a meshed split-thickness skin graft at postoperative day 14. **c** Photograph 1 year after muscle flap coverage. **d** Photograph 1 year after operation showing the functional limb salvage with ambulatory status

In the second operation, the exposed surface of the tibia was debrided by an orthopedic surgeon to completely remove the fragile bone tissue. Then, a saphenous vein and the free gracilis muscle with a pedicle were harvested from the same incision in the right thigh. The length and quality of the pedicle of the gracilis muscle were confirmed preoperatively by ultrasonography and CTA (Fig. 2c). An additional jump bypass was performed from a previous graft to the distal PTA through a subcutaneous tunnel made under intact skin. Then, the pedicle artery of the gracilis muscle was anastomosed with the additional graft and the pedicle vein was anastomosed with the posterior tibial vein by vascular surgeons (Fig. 3a). The muscle flap was inserted in front of the tibia and then covered with a meshed split-thickness skin graft that was harvested from the left thigh (Fig. 3b).

The patient’s postoperative recovery was uneventful; ulcers were completely epithelialized 5 months after the initial operation. Following the intravenous antibiotics for 4 months, oral antibiotics were continuously prescribed to prevent the recurrence of osteomyelitis: For 3 months, two antibiotics with minocycline hydrochloride and sulfamethoxazole trimethoprim were used, then reduced to one antibiotic with sulfamethoxazole trimethoprim for the next 1 year. After revascularization, walking rehabilitation was performed under the support of lower limb orthosis for drop foot while the weight load on the foot was gradually increased. After rehabilitation, lower limb muscle strength was sufficiently recovered. The patient was discharged 6 months after the initial surgery with ambulatory; no infection recurrence was observed after 1 year of follow-up (Fig. 3c, d), and even after 2 years, he maintained ambulatory function. Patient consent was obtained to publish this report.

Revascularization through bypass surgery or EVT plays a pivotal role in preventing limb loss and improves quality of life and survival of patients with CLI [3, 4]. Although bypass surgery has been the standard method for revascularization, EVT has recently become more popular because it is an advanced technology and is minimally invasive. However, in some cases of inappropriate and failed EVT, delay in definitive revascularization can result in a major amputation that is accompanied by deep infection [5]. In such cases, bypass surgery for limb salvage is sometimes quite demanding because of the extensive disease resulting from severe gangrene and infection involving the target arteries. Additionally, concomitant osteomyelitis makes it difficult to judge the likelihood of limb salvage. To salvage such limbs, vascular surgeons have to apply complicated and demanding revascularization strategies that will make the amputation level as low as possible. In the case described in this report, because of the extensive tissue loss and osteomyelitis caused by the failed EVT, the possibility of major amputation seemed to be high even after a successful bypass to the PTA. We decided to make dual bypasses to both the SA and PTA to avoid transfemoral amputation.

Bypass to the perigeniculate collateral arteries, including the SA, has previously been described as an option for limb salvage in selected patients, such as those with extensive artery disease, those in whom a previous revascularization has failed, or those who lack the usual crural arterial runoff [6, 7]. The aim of this operation is to increase blood flow through collateral vessels that are mainly nurtured by the perigeniculate arteries. This type of bypass grafting is quite exceptional and should be only used as the last resort when other conventional bypasses are impossible, and a bypass to a perigeniculate vessel alone may not be sufficient to allow tissue healing in patients with extensive foot necrosis [6, 7]. Therefore, we decided to apply an SA bypass in combination with revascularizing the PTA, expecting the perigeniculate artery bypass to reduce the amount of amputation required in a case in which a major amputation was unavoidable. Patent perigeniculate grafts may provide flow to the transtibial amputation level, enhancing the tissue healing capacity of the amputated surface, although a bypass graft to the PTA can be resected when a transtibial amputation is necessary.

In the present case, we used a gracilis muscle flap to cover the tissue loss area. The most frequently used free-muscle flaps for the lower extremities are the latissimus dorsi and rectus abdominis, and the gracilis muscle is usually a third choice [8–10]. However, the rather slim and long shape of the gracilis muscle was considered suitable for the defect at the front of the tibia in this case. Gracilis muscle is also easily accessed by vascular surgeons, even under regional anesthesia; the gracilis muscle is harvested from the same incision that is used for harvesting the great saphenous vein and thus does not require an intraoperative change of the patient’s position. However, the pedicle artery of the gracilis muscle may be affected by arteriosclerotic changes in patients with arteriosclerotic risk factors, and preoperative imaging analysis of the pedicle will be important to decide whether it is possible to use the gracilis muscle.

Reconstruction of soft tissue defects in the presence of chronic osteomyelitis remains difficult [11]. Even successful arterial reconstruction and free flap transfer does not necessarily guarantee the salvage of legs with extensive osteomyelitis [12], and adequate wound debridement and strict control of infection are prerequisites for the successful free flap insertion, prevention of infection recurrence, and subsequent amputation [10, 13]. The timing of tissue transfer will be also quite important to prevent infection recurrence, and in the present case, we decided the timing of the second surgery by the wound appearance with good granulation without active infection signs and well-controlled inflammation and osteomyelitis limited on the surface of the exposed tibia on MRI.

Salvaging a severely ischemic limb with osteomyelitis is quite demanding because of the difficulty in judging the possibility of limb salvage. In this case, we judged limb salvage to be possible on the basis of the wound appearance, wound bacterial culture, systemic inflammatory state, MRI findings, and consultation with an orthopedic surgeon; after intensive multidisciplinary treatment with surgical revascularization, wound management, free muscle flap insertion, and intensive rehabilitation, we managed to achieve functional limb salvage.

## Conclusions

Even in the threatened limb with extensive tissue loss and osteomyelitis, intensive and multidisciplinary treatment with staged revascularization, muscle transfer, and appropriate wound care achieved functional limb salvage.

## Abbreviations

ATA: Anterior tibial artery
CFA: Common femoral artery
CLI: Critical limb ischemia
CTA: Computed tomography angiography
DFA: Deep femoral artery
EIA: External iliac artery
EVT: Endovascular therapy
MRI: Magnetic resonance imaging
PTA: Posterior tibial artery
SA: Sural artery
SFA: Superficial femoral artery
